# Flexible mechanical metamaterials enabling soft tactile sensors with multiple sensitivities at multiple force sensing ranges

**DOI:** 10.1038/s41598-021-03588-y

**Published:** 2021-12-16

**Authors:** Alireza Mohammadi, Ying Tan, Peter Choong, Denny Oetomo

**Affiliations:** 1 Human Robotics Lab, Department of Mechanical Engineering, The University of Melbourne, Parkville, VIC 3010 Australia; 2 Australian Research Council Centre of Excellence for Electromaterials Science, Wollongong, NSW 2500 Australia; 3 Department of Surgery, University of Melbourne at St Vincent’s Hospital, Fitzroy, VIC 3065 Australia

**Keywords:** Mechanical engineering, Polymers, Composites, Sensors and biosensors

## Abstract

The majority of existing tactile sensors are designed to measure a particular range of force with a fixed sensitivity. However, some applications require tactile sensors with multiple task-relevant sensitivities at multiple ranges of force sensing. Inspired by the human tactile sensing capability, this paper proposes a novel soft tactile sensor based on mechanical metamaterials which exhibits multiple sensitivity regimes due to the step-by-step locking behaviour of its heterogenous multi-layered structure. By tuning the geometrical design parameters of the collapsible layers, each layer experiences locking behaviour under different ranges of force which provides different sensitivity of the sensor at different force magnitude. The integration of a magnetic-based transduction method with the proposed structure results in high design degrees of freedom for realising the desired contact force sensitivities and corresponding force sensing ranges. A systematic design procedure is proposed to select appropriate design parameters to produce the desired characteristics. Two example designs of the sensor structure were fabricated using widely available benchtop 3D printers and tested for their performance. The results showed the capability of the sensor in providing the desired characteristics in terms of sensitivity and force range and being realised in different shapes, sizes and number of layers in a single structure. The proposed multi-sensitivity soft tactile sensor has a great potential to be used in a wide variety of applications where different sensitivities of force measurement is required at different ranges of force magnitudes, from robotic manipulation and human–machine interaction to biomedical engineering and health-monitoring.

## Introduction

In recent years, there has been a great deal of interest in soft and flexible tactile sensors due to their inherent safety for interaction with humans and environment, flexibility for wearable devices, and ease of integration in applications such as soft robotic hands for dexterous object manipulation, smart prosthesis, and health monitoring^1–7^. As interactions in different environment get more complex in real-world applications, there is a strong need for tactile sensors which can accommodate for different interaction forces with different sensitivities. For instance, integrating different stages of the object manipulation, such as tactile object recognition, texture identification, slip detection and in-hand manipulation requires tactile sensors with different sensitivities at different force sensing ranges.

The existing soft tactile sensors generally only provide a single predetermined force sensing sensitivity and dynamic range depending on the stiffness of the force transfer material and sensing principles (resistive, capacitive, optical, etc.), for example, see^8,9^, and references therein. As every sensing principle has its own capacity limit, the trade-off between sensitivity and sensing range has been an intrinsic problem. Usually, the mechanical structures of these soft tactile sensors are carefully calibrated, leading to either high sensitivity across a narrow force sensing range or low sensitivity at a wide force sensing range^10–12^.

There have been some attempts to widen the force sensing range while keeping the sensitivity at a reliable level (simultaneously achieving both high contact sensitivity and wide force range) using hierarchical fabric-based resistive sensor^13^, intrafillable microstructures^14^, multilevel microstructures^15^ or flexible piezoresistive sensors based on pressure-peak effect^16^. However, the functionality of these sensors is still limited to specific sensitivity and force sensing range depending on the material composition or fabrication procedure. To the best of authors’ knowledge, there is no soft tactile sensor that can produce multiple arbitrary, pre-defined sensitivities and force sensing ranges.

Inspired by the human skin, which has multiple layers with different stiffnesses^8,9^, this paper proposes a heterogenous multi-layered structure and the associated design framework to produce a soft tactile sensor for multiple sensitivities across force sensing ranges. This is achieved through a multi-layered structure with tunable structural stiffness of each layer. The proposed structure will allow contact forces to be transduced into the displacement of the different layers at different rates, depending on the stiffness of the layers. Different rates of force–displacement curves are corresponding to different sensitivities of the resulting tactile sensor.

In order to realise a structure with multiple pre-defined and tunable force–displacement curves, flexible/soft mechanical metamaterials are exploited in this paper. Mechanical metamaterials are architectured structures with mechanical properties defined by their structure rather than their chemical composition^17–19^. Altering the topology of the mechanical metamaterials will result in novel mechanical properties, through the structural manipulation of known material(s). The desired properties of mechanical metamaterials can be achieved by exploring the ample design space offered by the geometrical parameters of their structure^20^. Different structures are used in realisation of soft sensing systems such as auxetic structure for stretchable strain sensor^21^, lattice-based structure for pressure sensing^22^, or origami-based structure for electromyography signal detection^23^.

In this paper, we propose a multi-layered cellular mechanical metamaterial as the force transfer structure of the soft tactile sensor that through changing the geometrical parameters of the structure, creates a large library of linear force–displacement (stiffness) patterns for the desired stiffness of each layer. Then, using a transduction method that can provide the measurement of the resulting displacement of the layers, the contact force can be measured with different sensitivities at different corresponding force sensing ranges. In this study, a magnetic field-based transduction method^24–26^ is used to measure the displacement of the layers. The changes in the measured magnetic field can provide the displacement information and in combination with the mechanical behaviour of each layer will provide the desired characteristics in terms of sensitivity and force sensing range.

## Results and discussions

### Mechanical metamaterial structure of the sensor

**Figure 1:**
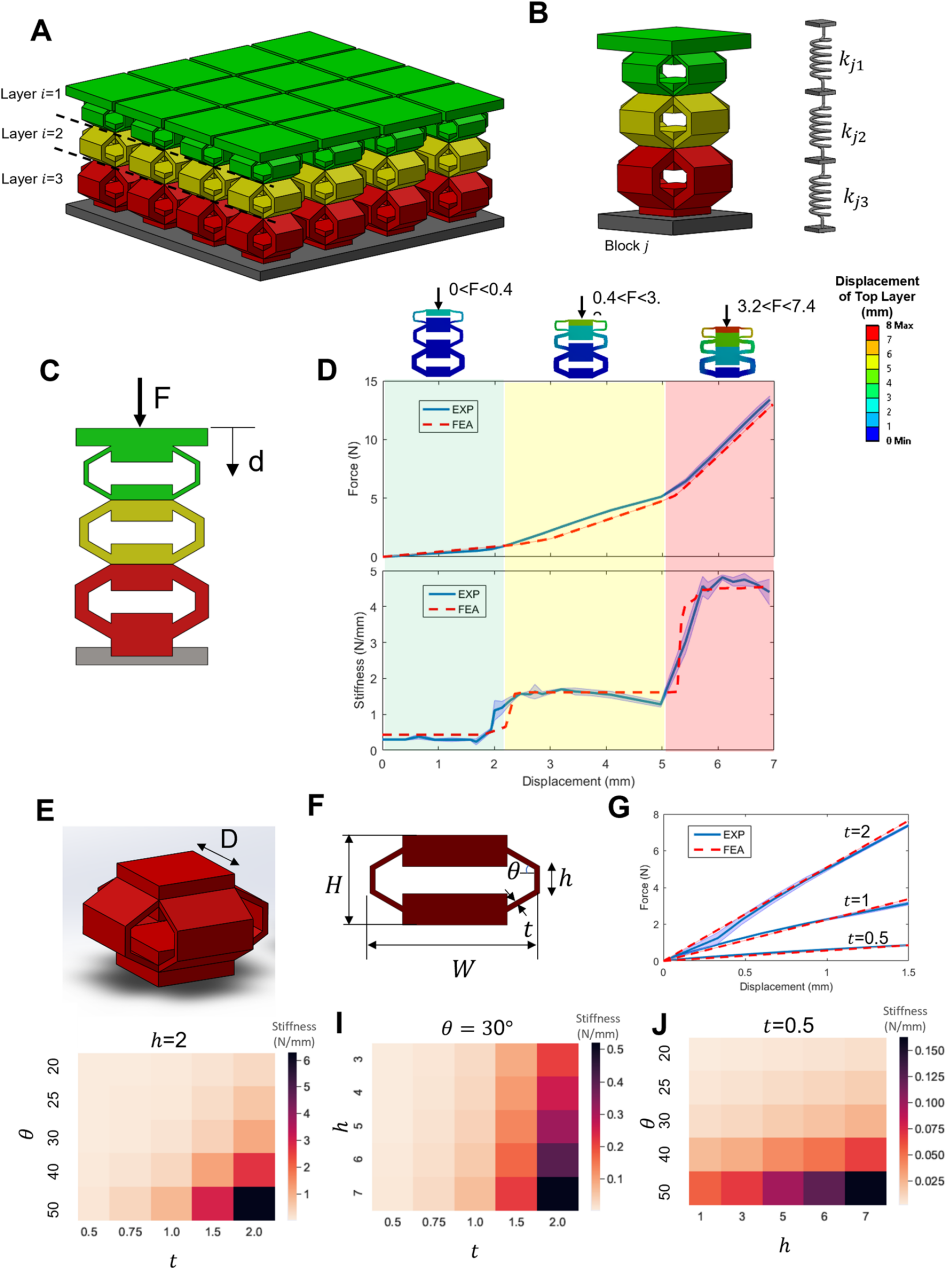
Mechanical metamaterial structure of the MST sensor force transfer medium. (**A**) Schematic of the MST sensor structure with an array of multi-layered blocks. (**B**) A single block schematic and its spring model analogous. (**C**) The cross-section of a single block with three layers. (**D**) Force–displacement plot of a single nominal block and its corresponding stiffness values. (**E**) 3D model of a unit cell. (**F**) Tuning parameters of a single unit cell ($\theta$, $t$, $h$) for a given geometrical dimension of the unit cell (Depth, $D$ – Height, $H$ – Width, $W$). (**G**) FEA and experimental results of a unit cell stiffness for different *t* values (**H-J**) Heatmap representation of the FEA results of the nominal unit cell stiffness for different set of tuning parameters.

Figure 1A shows the mechanical metamaterial structure of the force transfer medium of the proposed Multi-sensitivity Soft Tactile (MST) sensor. The mechanical metamaterial structure of the sensor is composed of an array of blocks (Fig. 1A) where each block $j$ consists of multiple stacked unit cells forming $i$ number of layers (Fig. 1B) and each unit cell $i$ can be considered as a spring with the stiffness ${k}_{ji}$*.* The size and number of the blocks can be altered based on the required size of the sensing area and the spatial resolution of the sensor. Only three layers are presented in the figure, but the number of layers, which depends on the desired sensitivities and corresponding force sensing ranges, can be varied.

The stiffness of each unit cell in the block is different depending on the geometrical structure and material properties of the unit cell (Fig. 1C) and they are stacked on each other in ascending order (the unit cell with the lowest stiffness is at top). Upon the force application, the unit at the first layer will move until it is locked to the next layer. Further increase of the applied force will result in translation of the first layer at different gradient corresponding to stiffness of the unit cell, as shown in Fig. 1D. This procedure will be continued till the last two layers are locked. This will result in a force–displacement curve with multiple linear regions corresponding to multiple stiffness values. With a good approximation, each of these stiffness values are corresponding to the stiffness of each constituent unit cell of the MST sensor structure if ${k}_{j1}\ll {k}_{j2}\ll \dots \ll {k}_{ji}$. Therefore, the overall desired stiffness variation can be achieved through altering the stiffness of each unit cell and, in turn, this can happen via changing their tuning parameters as discussed in the next subsection.

### Tuning parameters of unit cells

Each unit cell of the flexible mechanical metamaterial structure (Fig. 1E) has multiple parameters that can be tuned, leading to the desired stiffness of the unit cell. As shown in Fig. 1F, at a given geometrical dimension of the unit cell (Depth, $D$ – Height, $H$ – Width, $W$), there are 3 parameters ($\theta$, $t$, $h$) which affect the stiffness of the unit cell. For a structure with $j$ blocks and $i$ unit cells in each block, these parameters provide a large design space to achieve the desired stiffness of each unit cell of the MST sensor. As an example, Fig. 1G shows the force–displacement relation of a nominal unit cell ($D$ = 20 mm, $H$ = 5 mm, $W\hspace{0.17em}$= 30 mm) for three different values of tunning parameter *t* (0.5 mm, 1 mm, and 2 mm), and its effect on stiffness values. The result shows a good consistency between finite element analysis (FEA) simulations and experiments and also demonstrates a linear behaviour over the maximum displacement range. Therefore, stiffness of each unit cell, corresponding to a set of tunning parameters, can be represented with a single value. Figure 1H–J show that different combination of tuning parameters can result in a wide range of stiffness values from 0.02 to 6 N/mm in this nominal unit cell size.

### Magnetic-based transduction

In order to be able to obtain the applied force on the sensor using the force–displacement curves, we need to measure the displacement of the top layer with respect to the base layer of the MST sensor. There are different transduction methods, such as resistive, capacitive, or magnetic, to convert the displacement of the MST sensor layers into electric signals. In this paper, we exploit the magnetic-based method due to its advantages including no physical connection between permanent magnet stimuli at top layer and magnetometer at the base layer, high sensitivity of the magnetometer to the changes in the magnetic field, and a linear response ^8^.

**Figure 2:**
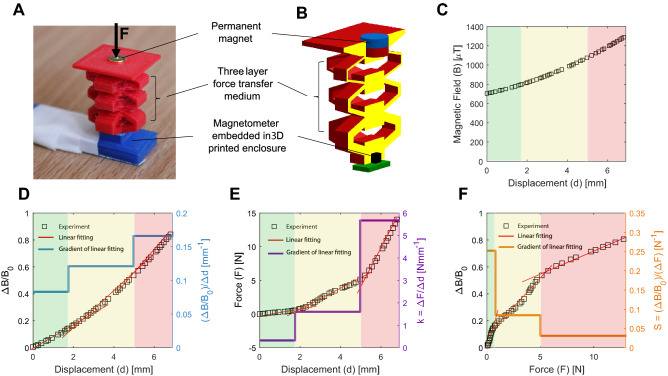
Magnetic-based transduction method of the MST Sensor. (**A**) A single three-layer block with integrated permanent magnet as stimulus, and magnetometer covered by a 3D printed holder. (**B**) Schematic cross-section of the single block. (**C**) The variation of the overall magnetic field with respect to the displacement of the permanent magnet in the top layer. (**D**) The variation of the $\Delta B/{B}_{0}$ with respect to the permanent magnet displacement (left y-axis), linear fitting (red lines) at three locking stages (highlighted background in green, yellow, and red), and gradient of linear fitting (right y-axis) corresponding to $(\Delta B/{B}_{0})/\Delta d$. (**E)** The variation of the applied force with respect to the displacement of top layer (left y-axis) linear fitting (red lines) at three locking stages, and gradient of linear fitting (right y-axis), corresponding to the stiffness of different layers of the block ($k=\Delta F/\Delta d$). (**F**) The variation of the $\Delta B/{B}_{0}$ with respect to the applied force (left y-axis), linear fitting (red lines) at three locking stages, and gradient of linear fitting (right y-axis), corresponding to the multiple sensitivities of the MST sensor at different force sensing ranges ($S=(\Delta B/{B}_{0})/\Delta F$).

The integration of the magnetic-based method with the metamaterial structure is shown in Fig. 2A and its cross-section representation in Fig. 2B. In this single block, a permanent magnet is embedded at the top layer of the block and a magnetometer at the base of the block. Upon applying the force, the position of the permanent magnet will change with respect to the magnetometer. This results in changes in the measured magnetic field and can provide the displacement information. Combining the magnetic field-displacement information with force–displacement curves, we can obtain the applied normal force magnitude or pressure value. The relation between the applied force and magnetic field can be calibrated with respect to any of the magnetic field components or the overall magnetic field as established in the literature^24–26^. Figure 2C shows the variation of the overall magnitude of the magnetic field ($B$) with respect to the displacement of top cell ($d$) for the example design of Fig. 2A. The variation of the magnetic field components is shown in Fig. S1. The gradient of changes of the magnetic field with respect to the applied force and the corresponding displacement of the metamaterial layers indicates the sensitivity of the MST sensor as explained the next subsection.

### Sensitivity and force sensing range

Sensitivity is defined as the variation in the measured magnetic field magnitude with respect to the contact force magnitude and can be formulated as:

1 $$S=\frac{(\Delta B/{B}_{0})}{\Delta F} \quad[{\text{N}}^{-1}]$$

where $\Delta F$ is the variation in the contact force, ${B}_{0}$ is the initial measured magnetic field without any applied force, and $\Delta B$ is the corresponding change in the magnetic field. Considering a single block of the MST sensor with $i$ unit cells and the $i$th unit cell as a spring with stiffness $k_{i}$ and displacement ${\Delta }d_{i}$, then the sensitivity of the $i{\text{th}}$ unit cell can be represented as:

2 $$ S_{i} = \frac{{\left( {{\Delta }B/B_{0} } \right)/{\Delta }d_{i} }}{{k_{i} }} \quad\left[ {{\text{N}}^{ - 1} } \right] $$

at the force sensing range of the $i$th unit cell which is considered as:

3 $$ R_{i} = {\Delta }d_{i}^{max} \times k_{i} \quad\left[ {\text{N}} \right] $$

where $\Delta d_{i}^{max}$ is the maximum displacement of the $i{\text{th}}$ unit cell before locking to the next unit cell.

The Eqs. (1)–(3) allows the tunning the MST sensor design parameters to achieve multiple sensitivities at multiple force sensing ranges. In these equations, ${\Delta d}_{i}^{max}$ and ${k}_{i}$ are related to the size and stiffness of the unit cells of the mechanical metamaterial structure, which can be altered through their geometrical parameters including ${D}_{i}, {H}_{i},{W}_{i},{\theta }_{i},{t}_{i},{h}_{i}$, as defined in Fig. 1E,F. In addition, the variation of the magnetic field with respect to the displacement of the unit cells, $(\Delta B/{B}_{0})/\Delta {d}_{i}$, depends on the permanent magnet characteristics (size and magnetisation) and magnetometer sensitivity (${S}_{M}$). All these parameters are affecting the sensitivity and force sensing range of the MST sensor. For instance, in the example design of Fig. 2A, with more details in the Supplementary Text, the variation of the magnetic field with displacement of the permanent magnet at top layer (Fig. 2D) can be approximated with three ($i$=1,…,$n$ and $n$=3) linear regions corresponding to three linear regions of the force–displacement curve in Fig. 2E, resulted from three unit cells with different stiffness values. This results in three sensitivities (0.25, 0.08, 0.03)${N}^{-1}$ at three corresponding force ranges (0.9, 4.8, 16.2) $N$ of the MST sensor as represented in Fig. 2F.

Nevertheless, we cannot arbitrarily choose the parameters to achieve the desired characteristics due to the trade-off between different parameters, constraints on the size of the sensor, and functional limitations of the magnetometer. Therefore, a design framework is required to systematically consider the trade-offs and constraints to achieve the desired characteristics of the MST sensor.

### MST sensor design framework

**Figure 3:**
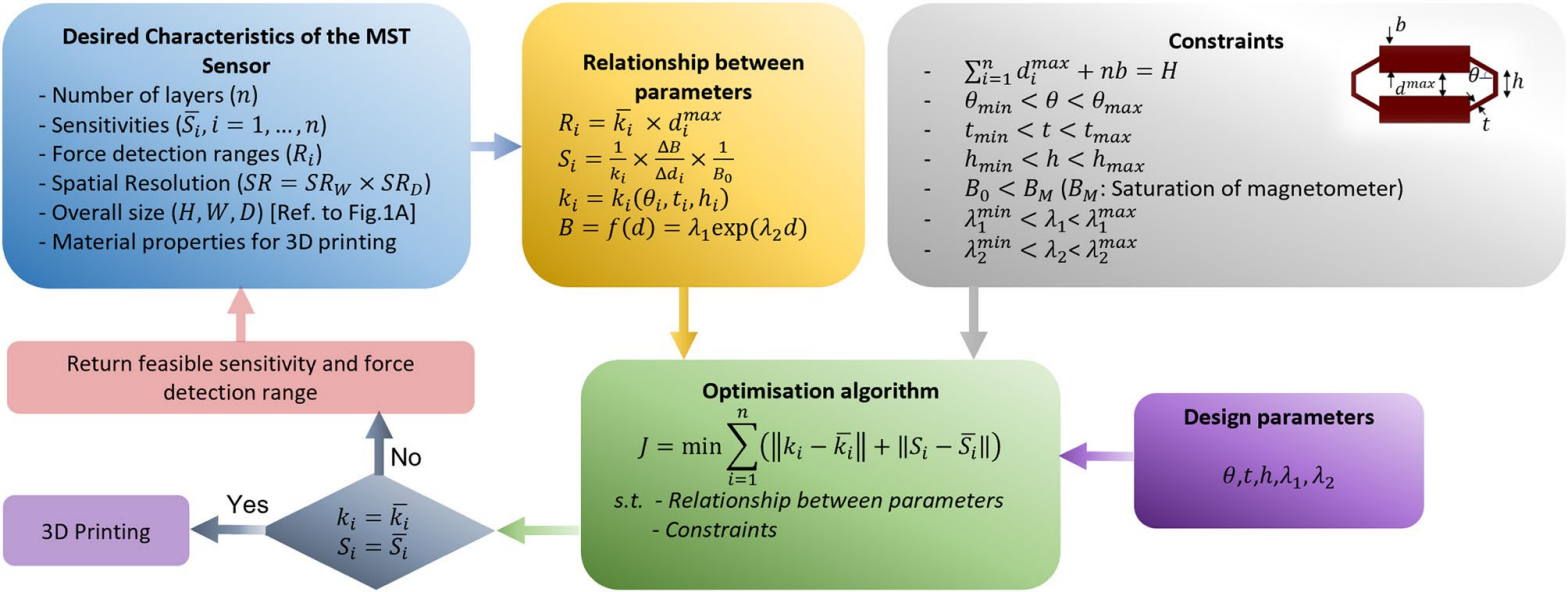
The design framework of the MST sensor. Given the desired characteristics of the sensor, this design procedure will provide the design parameters of the sensor structure.

The objective of this systematic design framework is to determine the values of the design parameters for fabrication of the MST sensor based on the desired characteristics. The block diagram of the proposed design framework is shown in Fig. 3. The blue rectangle in Fig. 3 shows the given desired characteristics of the MST sensor including the overall size and spatial resolution which specifies the number of required blocks in the mechanical metamaterial structure. In addition, the number of layers or unit cells ($n$) in each block and their corresponding desired sensitivities and force sensing ranges are given. The yellow rectangle represents the relation between the sensitivity and force sensing range and design parameters as defined in Eqs. (1)–(3). Moreover, the relation between the magnetic field measurement in the magnetometer, $B$, and the displacement, $d$, of the permanent magnet embedded in the top layer can be defined as function $f$. In most cases, the function $f$ can be represented with an exponential function as $f={\lambda }_{1}\mathrm{exp}({\lambda }_{2})$ with two constant parameters, ${\lambda }_{1}, {\lambda }_{2}$. The values of these parameters depend on the size and magnetic characteristics of the permanent magnet.

The available design parameters (the purple rectangle in Fig. 3) are ${\theta }_{i}, {t}_{i}, {h}_{i}, {d}_{i}^{max},{\lambda }_{1},{\lambda }_{2}$ which specifies the geometrical dimensions of the ${i}$th unit cell of a block with $n$ unit cells or layers ($i$ = 1,…,$n$) and also the behaviour of magnetic field variation with respect to the displacement of the permanent magnet. The spatial resolution and size of the MST sensor will impose constraints on the available range of the design parameters (the grey rectangle in Fig. 3). The other constraints are related to the limitations of the magnetometer regarding magnetic saturation and sensitivity and the size of the permanent magnet that can be embedded in the top layer.

Since there are multiple design parameters affecting the sensitivity and force sensing range of the MST sensor and each design parameter has different effect on characteristics of the sensors, an optimisation algorithm is required to find a set of parameters that provides desired characteristics. To this end, we define a cost function that its minimisation will result in desired sensitivities and stiffness values of each layer (corresponding to force sensing range) and will provide the values of design parameters. This optimisation algorithm requires the stiffness model with respect to the geometrical design parameters of the mechanical metamaterial structure, $k=k\left(\theta ,t, h\right)$, which can be modelled using FEA results and then implementing the regression models (more details in the Supplementary Text).

The design parameters obtained from optimisation algorithm determines the mechanical metamaterial structure of the force transfer medium of the MST sensor and the resultant structure can be fabricated through additive manufacturing using standard 3D printers. Due to the imposed constraints, there are cases where there is no feasible solution for the optimisation algorithm. In these cases, the algorithm will return the maximum feasible sensitivities and force sensing ranges to the designer to either relax the constraints or provide a revised desired characteristics.

### Performance demonstration

**Figure 4:**
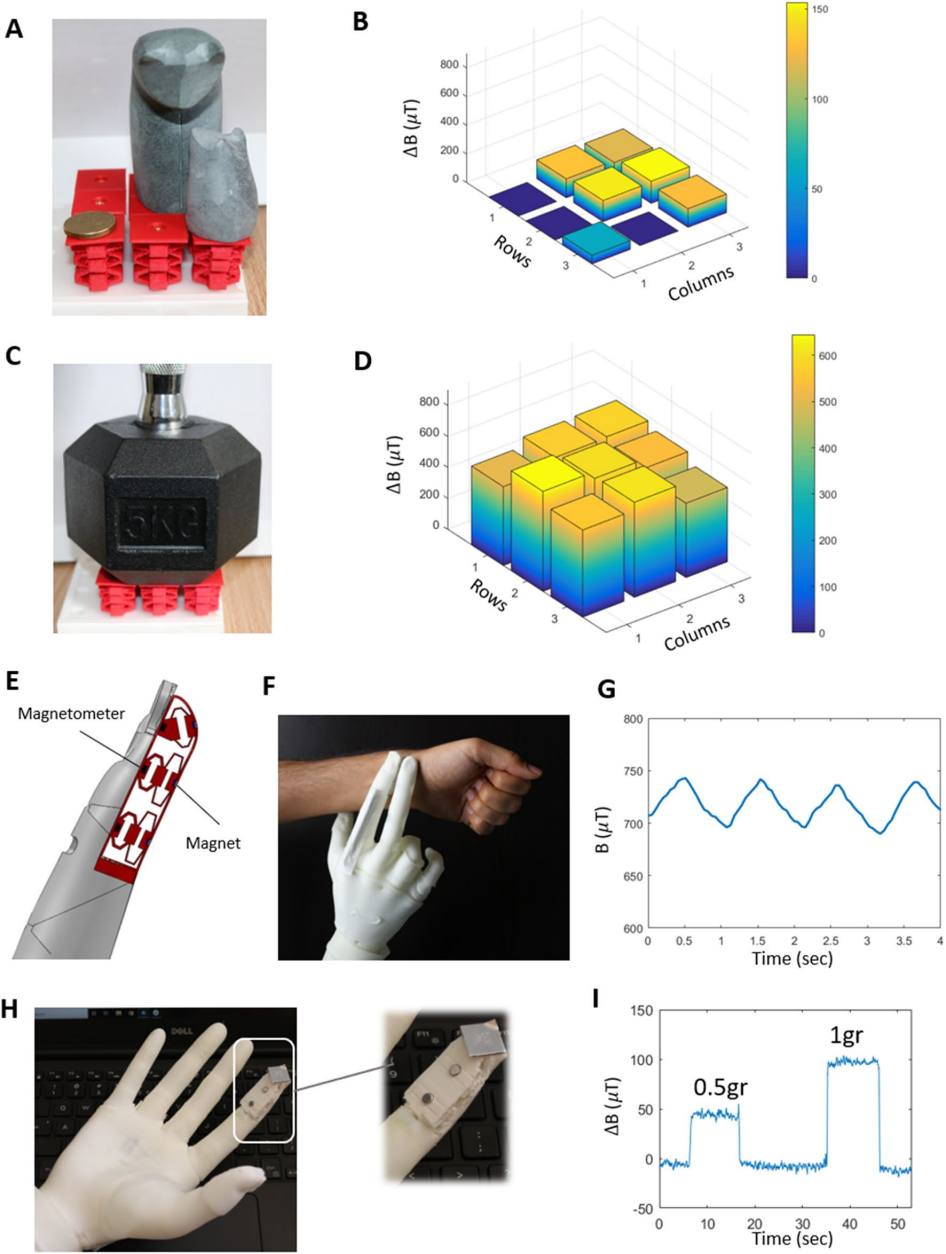
Performance demonstration of two example designs of the MST sensor. (**A**) A $$3\times 3$$ array of three-layer blocks with three objects on them: a $2 Australian coin (6.6gr), a handicraft stone kitten (56gr), and cat (265gr). (**B**) Results of the MST sensor reading of the coin and handicraft stone kitten and cat. (**C**) A 5 kg dumbbell on the MST sensor array. (**D**) Results of the sensor reading of the 5 kg dumbbell. (**E**) The schematic of the robotic prosthetic finger with integrated MST sensor. (**F**) 3D printed robotic prosthetic hand with embedded MST sensor in its index finger for monitoring the pulse of human radial artery. (**G**) The magnetic field variation of the middle block of the MST sensor due to the pulse wave. (**H**) The robotic hand with integrated MST sensor for detecting small contact forces. (**I**) The response of the highly sensitive unit cell at the tip of the finger to different weights.

In order to demonstrate the performance of the proposed MST sensor, two different designs are constructed and tested. In the first design, we fabricated a $$3\times 3$$ pixel array which includes 9 blocks with three layers. The desired sensitivity and force sensing range for each layer of these blocks are set to (0.2, 0.1, 0.01) $${N}^{-1}$$ and (0.5, 5, 15) $$N$$, respectively. The maximum size of each block is considered as 20 mm $$\times $$ 20 mm with no constraint on height. A disc-shape Neodymium gold coated magnet with diameter of 6 mm and thickness of 2 mm and remanent magnetisation of 1.3 T is used as a stimulus. The tri-axis magnetometer (MLX90393, Melexis Inc.) used to measure the magnetic field in three directions with the sensitivity of 6211 mT^−1^ and resolution of 0.161 μT. The combination of the permanent magnet and magnetometer results in relation between the magnetic field–displacement relation of $$B=f\left(\mathrm{d}\right)={\uplambda }_{1}\mathrm{exp}({\lambda }_{2}d)$$ with $${\uplambda }_{1}=140$$ and $${\lambda }_{2}=0.2$$. The stiffness model, $$k=k\left(\theta ,t, h\right)$$, is obtained through implementing the multi-variable neural network regression model of the MATLAB software (MathWorks Inc.) on the FEA results of Fig. 1H–J. In the next step, the Direct Pattern Search algorithm of the MATLAB is used to find the design parameter values of the mechanical metamaterial structure. Details of the implementation of the proposed design framework in finding the structural parameters of this design is provided in the Supplementary Text. The obtained parameters are $$\theta = \left( {25^\circ ,33^\circ ,37^\circ } \right), t = \left( {0.6,1,1.4} \right)\;{\text{mm}}, \;{\text{and}}\; h = \left( {2,2.8,3.5} \right)\;{\text{mm}}$$ for three unit cells of the first design. These design parameters result in the sensitivity and force range of (0.26, 0.08, 0.03) N^−1^ and (0.9,4.8,16.2) N, respectively, which are close to the desired characteristics. The structure is fabricated using 3D printing of Thermoplastic Polyurethane (TPU) and the permanent magnet and magnetometer are then integrated in the structure. The performance of the first MST sensor design is demonstrated in sensing objects with the weight in the range from 6gr ($$\approx $$ 0.06 N) to 5000gr ($$\approx $$ 50 N), as shown in Fig. 4A–D and Movie S1. Figure 4A and 4B show the sensitivity of the sensor in measuring object weight at the light end, such as the $2 Australian coin (6.6gr) and the handicraft stone kitten (56gr). The same sensor can also be used to measure the weight of an object at the 5000 g range, as shown in Fig. 4C and 4D.

The purpose of the second design is to show that the MST sensor can be realised in different shapes, sizes, and different number of layers in a single structure. As shown in Fig. 4E, a specific mechanical metamaterial structure of the MST is integrated in the fingertip of a 3D printed soft robotic prosthetic hand^27^. This MST sensor design has two layers at the base and a single layer with highly sensitive unit cell at the tip. The performance of the two-layer base structure is demonstrated in sensing the pulse of the human radial artery (Fig. 4F and Movie S2). The magnetic field variation of the middle block of the MST sensor due to the pulse wave is shown in Fig. 4G. The response of the highly sensitive unit cell at the tip of the finger (Fig. 4H) to the weights as small as 0.5gr is shown in Fig. 4I and demonstrated in Movie S3.

The results showed that the MST sensor can be designed and fabricated in a wide variety of shapes and sizes*.* Depending on the application, the building blocks of the sensor can also have different mechanical metamaterial with different number of layers. The blocks of the sensor can be isolated from each other (e.g., Fig. 4A) or interconnected in one or more of the layers (e.g. Figure 4E). In the case that the blocks are interconnected, the effect of interconnection should be considered in the calculation of the stiffness. The effect of the interconnected blocks on stiffness is demonstrated through an illustrative example in Fig. S2.

In addition to all the geometric design parameters, the material properties of the sensor force transfer medium for 3D printing should be considered as one of the key parameters in the design procedure. Although the proposed MST sensor can be fabricated with a single material, there is no limitation in using multiple materials in realisation of the sensor. With the advances in multi-material 3D printing, fabrication of objects with multiple materials are getting more available and cost effective. The availability of fabrication of the MST sensor with multi-material offers extra dimensions and flexibility in the design procedure.

## Conclusions

This study presented a novel and easy-to-manufacture soft tactile sensor using flexible mechanical metamaterials in its force transfer structure with the capability for realising multiple desired sensitivities at arbitrary force sensing ranges. This is possible through a large design space provided by design parameters of the metamaterial structures in addition to magnetometer sensitivity, permanent magnet characteristics, and the selected material for 3D printing. The fabrication of the MST sensor with standard benchtop 3D printers significantly simplifies its manufacturing and its integration in different robotic systems. The two example designs of the MST sensor in this study demonstrates its great potential to be used in wearable diagnostic device for human health monitoring, future care robots and domestic robots.

## Materials and methods

### Sensor fabrication

The material used for 3D printing of the sensor is Thermoplastic Polyurethane (TPU) with Shore hardness A85. For the 3D printing of TPU, the commercially available 3D printer (FlashForge Dreamer) was used. The 3D printer used in this paper (FlashForge) has an open-source software (FlashPrint) for slicing the STL files and preparing the file for 3D printing. In this software, by choosing the printing material, the extrusion speed and travel speed will be set automatically to an appropriate value. In our case “Flexible Filament” is used as Material Type which has the extrusion speed of 60 mm/s and travel speed of 80 mm/s. The nozzle temperature was set to 220 °C which is the recommended value by TPU filament (FilaForm) manufacturer, considering the glass transition temperature and melting temperature of TPU at − 44 °C and 195 °C, respectively.

### Force–displacement simulation and experiment

The force–displacement (stiffness) analysis carried out through the ANSYS software. Force–displacement experiments were conducted with Mecmesin force testing machine (Mecmesin Ltd, England).

### Sensor readout

The magnetometer was MLX90393, tri-axis magnetic sensors from Melexis Inc., providing magnetic field measurements in three axes thorough I2C fast mode protocol. Three MLX90393 magnetometers are connected to each other on a flexible printed circuit board (fPCB) and the signals can be read through a single channel I2C. One multiplexer (TCA9548A) with eight channels is used to connect three strips of three-magnetometer resulting in $3\times 3$ pixel array. The output of the multiplexer was fed to the microcontroller (ESP32, Espressif Systems). The microcontroller was connected through a serial port into a computer where magnetic field readings from magnetometers are acquired and visualised through MATLAB (MathWorks Inc.). The entire 9 readings were sampled four times per second. A form of moving average filter was used to smoothen the data. The same strip of magnetometers is integrated in the 3D printed soft robotic hand.

## Supplementary Information

Supplementary Information.

Supplementary Video 1.

Supplementary Video 2.

Supplementary Video 3.
