# Supplementary material for: Flexible mechanical metamaterials enabling soft tactile sensors with multiple sensitivities at multiple force sensing ranges
Source: Sci Rep. 2021 Dec 16;11:24125. doi: 10.1038/s41598-021-03588-y (PMC8677735; doi:10.1038/s41598-021-03588-y)
Supplement: Supplementary file 1 — Supplementary Information. [file 41598_2021_3588_MOESM1_ESM.docx]

**Supplementary Information**

**Flexible Mechanical Metamaterials Enabling Soft Tactile Sensors with Multiple Sensitivities at Multiple Force Sensing Ranges**

Alireza Mohammadi^1,2*^, Ying Tan^1^, Peter Choong^2,3^, Denny Oetomo^1,2^,

^1^Human Robotics Lab, Faculty of Engineering and Information Systems, The University of Melbourne, Parkville VIC 3010, Australia.

^2^Australian Research Council Centre of Excellence for Electromaterials Science, Wollongong, NSW 2500, Australia

3Department of Surgery of University of Melbourne at St Vincent's Hospital, Fitzroy, VIC 3065, Australia

^*^Correspondence to alirezam@unimelb.edu.au

**Supplementary Text**

This Text provides the details of implementation of the proposed design framework (Fig.3 in the manuscript) in finding the structural design parameters of the multi-sensitivity soft tactile (MST) sensor design which consists of a $3\times3$ pixel array with three layers. The desired sensitivity and force sensing range for each layer of these blocks are set to (0.5,0.2,0.1) $N^{-1}$ and (0.5,5,15) $N$, respectively.

In the first block of the design framework of Fig.3, the desired properties of the MST sensor are listed. The desired characteristics are as follows:

- Number of layers ($n=3$)
- Sensitivity of each layer: $\bar{S}_{1}=0.5$, $\bar{S}_{2}=0.2$, $\bar{S}_{3}=0.1$ [$N^{-1}$]
- Force sensing ranges: $\bar{R}_{1}=[0,0.5]$, $\bar{R}_{2}=\left[ 0.5,5 \right]$, $\bar{R}_{3}=[5,15]$ [$N$]
- Spatial resolution: $SR=SR_{w}\times SR_{D}=3\times3=9$
- Overall size: $W=60mm, D=60mm$ and no constraint on the height
- Material properties for 3D printing: Thermoplastic Polyurethane (TPU) with Shore hardness 85 (Young’s modulus *E* = 9.5 MPa and Poisson’s ratio *ν* = 0.38)

In the next block (yellow block of Fig.3) the relation between different parameters is provided. As we used a disc-shape Neodymium gold coated magnet with diameter of 6mm and thickness of 2mm and remanent magnetisation of 1.3T, the relation between the magnetic field and displacement of the permanent magnet can be represented as $B=f\left( d \right)=\lambda_{1}exp(\lambda_{2}d)$ with $\lambda_{1}=140$and $\lambda_{2}=0.2$. This is obtained from the experiments explained in the Material and Methods section of the manuscript. In addition, the relation between the stiffness and geometrical design parameters of the mechanical metamaterial structure, $k=k\left( \theta,t, h \right)$, is established using FEA results and then implementing the multi-variable neural network regression model of the MATLAB software (MathWorks Inc.) which is based on limited-memory Broyden-Flecter-Goldfarb-Shanno quasi-Newton algorithm (LBFGS).

The grey block of Fig.3 lists the constraints on design parameters and dimensions of the MST sensor. In this design the constraints are as follows:

- $b=2mm, d_{i}^{max}=3mm$
- $10^{\circ}<\theta<80^{\circ}$
- $0.5mm<t<2mm$
- $0.5mm<h<4mm$

The design parameters are $\theta$,$t$,$h$ as we assumed that the permanent magnet is given so $\lambda_{1},\lambda_{2}$ are fixed, as mentioned above. With the consideration of the desired characteristics of the MST sensor, relationship between parameters, constrains, and design parameters, we need to solve the following optimisation problem:

$$J=\min\sum_{i=1}^{3} \left( \left\| k_{i}-\bar{k}_{i} \right\|+\left\| S_{i}-\bar{S}_{i} \right\| \right)$$

| subject to: | $\bar{k}_{i}=\frac{\bar{R}_{i}}{d_{i}^{max}}$ for $i=1,2,3$ and $\bar{R}_{1}=0.5$, $\bar{R}_{2}=5$, $\bar{R}_{3}=15$ |
| --- | --- |
|  | $k_{i}=k_{i}\left( \theta_{i},t_{i}, h_{i} \right)$ for $i=1,2,3$ |
|  | $S_{i}=\frac{1}{k_{i}}\times$ $\frac{\Delta B}{\Delta d_{i}}$ $\times\frac{1}{B_{0}}$ for $i=1,2,3$ and $B_{0}=700\mu T$ |
|  | $\bar{S}_{1}=0.5$, $\bar{S}_{2}=0.2$, $\bar{S}_{3}=0.1$ [$N^{-1}$] |
|  | $B=f\left( d \right)=\lambda_{1}exp(\lambda_{2}d)$ with $\lambda_{1}=140$and $\lambda_{2}=0.2$ |
|  | $10^{\circ}<\theta<80^{\circ}$ |
|  | $0.5mm<t<2mm$ |
|  | $0.5mm<h<4mm,$ |

where in the cost function, $J$, the design parameters, ($\theta_{i}$,$t_{i}$,$h_{i}$), are affecting $k_{i}$ and $S_{i}$. The aim of this optimization algorithm is to find design parameters that can provide the closest stiffnesses and sensitivities to the required stiffness of the unit cells in different layers ($\bar{k}_{i}=d_{i}^{max}\bar{R}_{i}$) and desired sensitivities ($\bar{S}_{i}$). To this end, we solved the optimization problem using Direct Pattern Search algorithm of MATLAB with bounds applied based on the constraints. This results in the design parameters for layers 1-3 as:

$$\theta_{1}=25^{\circ}, t_{1}=0.6mm, h_{1}=2mm$$

$$\theta_{2}=33^{\circ}, t_{2}=1mm, h_{2}=2.8mm$$

$$\theta_{3}=37^{\circ}, t_{3}=1.4mm, h_{3}=3.5mm,$$

and these design parameter values provide the following sensitivities and force sensing rages:

$$S_{1}=0.26N^{-1}, S_{2}=0.08N^{-1},S_{3}=0.03N^{-1}$$

$$R_{1}=0.9N, R_{2}=4.8N, R_{3}=16.2N,$$

which are close to the desired sensitivities and force ranges at:

$$\bar{S}_{1}=0.2N^{-1},\bar{S}_{2}=0.1N^{-1},\bar{S}_{3}=0.01N^{-1}$$

$$\bar{R}_{1}=0.5N, \bar{R}_{2}=5N, \bar{R}_{3}=15N.$$

**Supplementary Figures**


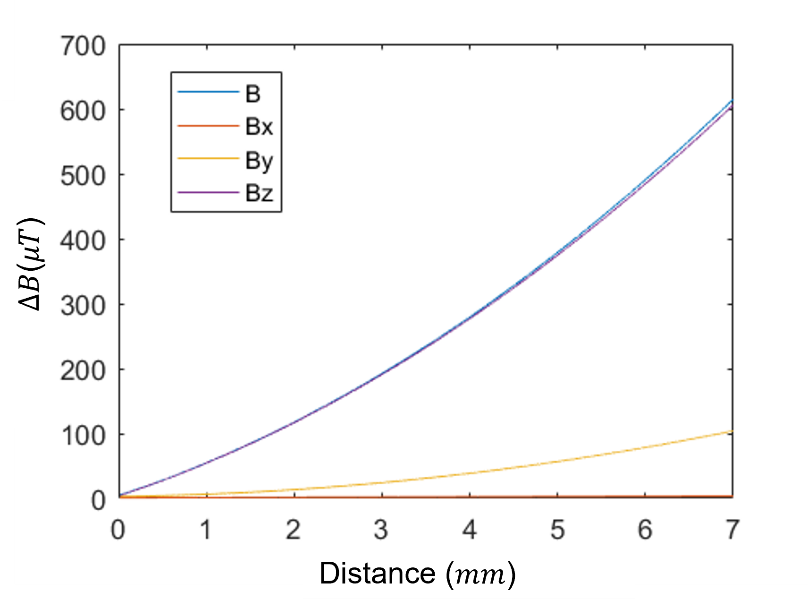


**Fig.S1.** Variation of the overall magnitude of the magnetic field and its components with respect to the displacement of top cell with an embedded permanent magnet.


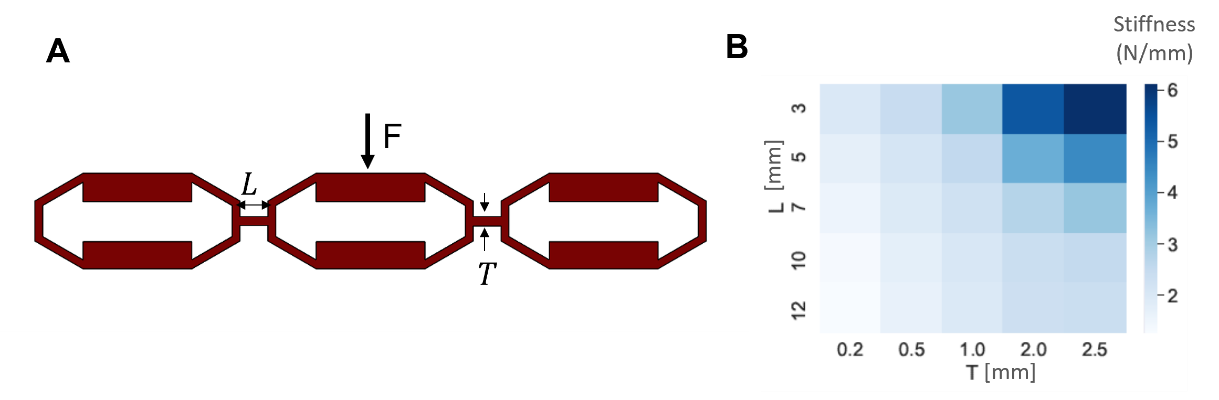


**Fig.S2. Interaction between unit cells.** **(A)** A mechanical metamaterial structure with three interconnected cells. **(B)** FEA results of the effect of length, L, and thickness, *T*, of the interconnection bridge on the stiffness of the middle cell.

**Supplementary Movies**

**Movie S1.** Performance of the MST sensor with a $3\times3$ pixel array in sensing the forces in the range from 6gr to 5000gr

**Movie S2.** Monitoring the pulse of the human radial artery with the MST sensor integrated in the fingertip of a 3D printed soft robotic prosthetic hand

**Movie S3.** Performance of a highly sensitive unit cell of the MST sensor integrated at the tip of the finger of a 3D printed soft robotic prosthetic hand
